# Paragonimiasis in tuberculosis patients in Nagaland, India

**DOI:** 10.3402/gha.v9.32387

**Published:** 2016-09-23

**Authors:** Mrinalini Das, Katerina Doleckova, Rahul Shenoy, Jagadish Mahanta, Kanwar Narain, K. Rekha Devi, Tongmeth Konyak, Homa Mansoor, Petros Isaakidis

**Affiliations:** 1 Médecins Sans Frontières (MSF), Delhi, India; 2 Médecins Sans Frontières (MSF), Mon, Nagaland, India; 3 Department of Biological Chemistry, The Alexander Silberman Institute of Life Science of HUJI, Jerusalem, Israel; 4 Regional Medical Research Centre (RMRC), Dibrugarh, Assam, India; 5 Médecins Sans Frontières, Operational Research Unit, Luxembourg City, Luxembourg

**Keywords:** lung fluke, northeast, zoonotic infection, praziquantel, operational research

## Abstract

**Background:**

One of the infections that mimic tuberculosis (TB) is paragonimiasis (PRG), a foodborne parasitic disease caused by lung flukes of the genus *Paragonimus*. In the northeastern states of India, TB and PRG are endemic; however, PRG is rarely included in the differential diagnosis of TB.

**Objective:**

To address limited evidence on the dual burden of TB and PRG in northeastern India, we aimed to document the prevalence of PRG among TB patients using sputum smear, stool examination for children <15 years and ELISA.

**Design:**

A cross-sectional study of patients receiving TB treatment in the Médecins Sans Frontières (MSF)-supported TB programme in Mon district, in collaboration with the Regional Medical Research Centre (RMRC), Dibrugarh, Assam, between November 2012 and December 2013.

**Results:**

Of 96 patients screened between November 2012 and December 2013, three (3%) had pulmonary PRG and were successfully treated with praziquantel.

**Conclusions:**

PRG should be considered in the TB diagnostic algorithms in PRG–TB dual burden areas. In case of TB–PRG co-infection, it is preferable to treat PRG first followed by anti-TB treatment a few days later.

## Introduction

Tuberculosis (TB) is one of the most ancient communicable diseases, ranked as the leading cause of death among infectious disease, along with HIV/AIDS. In 2014, an estimated 9.6 million people developed TB and 1.5 million died from the disease (1). The high mortality and morbidity of TB, especially multidrug-resistant TB, led to reinforced efforts to fight the TB pandemic, including policy discussions around sustainable developmental goals (2) and End TB Strategy (3). These initiatives are being incorporated into national and international policies and strategies. The TB clinical algorithms are being revised in most affected countries to reflect new tools allowing accurate diagnosis, new drugs and treatment regimens. However, accurate diagnosis continues to be challenging as TB is a multisystemic disease with myriad manifestations; it may masquerade as many other diseases or conditions and thus differential diagnosis based on the local epidemiology is essential.

One of the infections that mimic TB is paragonimiasis (PRG), a parasitic disease caused by lung flukes of the genus *Paragonimus* spp. (4). Both diseases are similar in clinical presentation including chronic cough, dyspnoea, haemoptysis and chest pain; however, the mode of transmission is different. TB is an airborne infection, and PRG is a food-borne trematode infection, usually caused after consumption of raw, pickled or insufficiently cooked freshwater crustaceans (crabs and crayfish) containing encysted metacercariae of the parasite. PRG is considered a rare and rather unusual condition of limited public health importance, and thus, it is widely neglected (5). The microscopic examination for PRG is cumbersome and has low sensitivity (6), making the PRG diagnosis extremely challenging in resource-limited countries. In the northeastern states of India, both TB and PRG have been present, and several endemic foci have been discovered (3, 4, 6). The prevalence of PRG ranged from 7 to 15% (3, 4) in the general population and around 50% in TB patients (7); however, PRG is often underdiagnosed.

Misdiagnosis of PRG may not only delay the initiation of appropriate treatment but also pose an unnecessary burden of long and toxic TB treatment on the patient (8). Serologic testing for PRG with high specificity has been previously found effective in population based screening in India (9). The current recommended regimen for PRG is a 3-day short course with praziquantel (10). In cases of TB–PRG co-infection, TB treatment failure may be wrongly diagnosed if lung symptoms persist.

Between 2010 and 2014, Médecins Sans Frontières (MSF)/Doctors Without Borders had collaborated with Mon District Hospital, Nagaland, in providing integrated care to TB patients (11). Based on empirical observations of relatively high numbers of negative acid-fast bacilli sputum smears among patients with TB and based on the local epidemiology, we hypothesised that PRG may be included in the programme's TB diagnostic algorithm.

## Methods

**Fig. 1:**
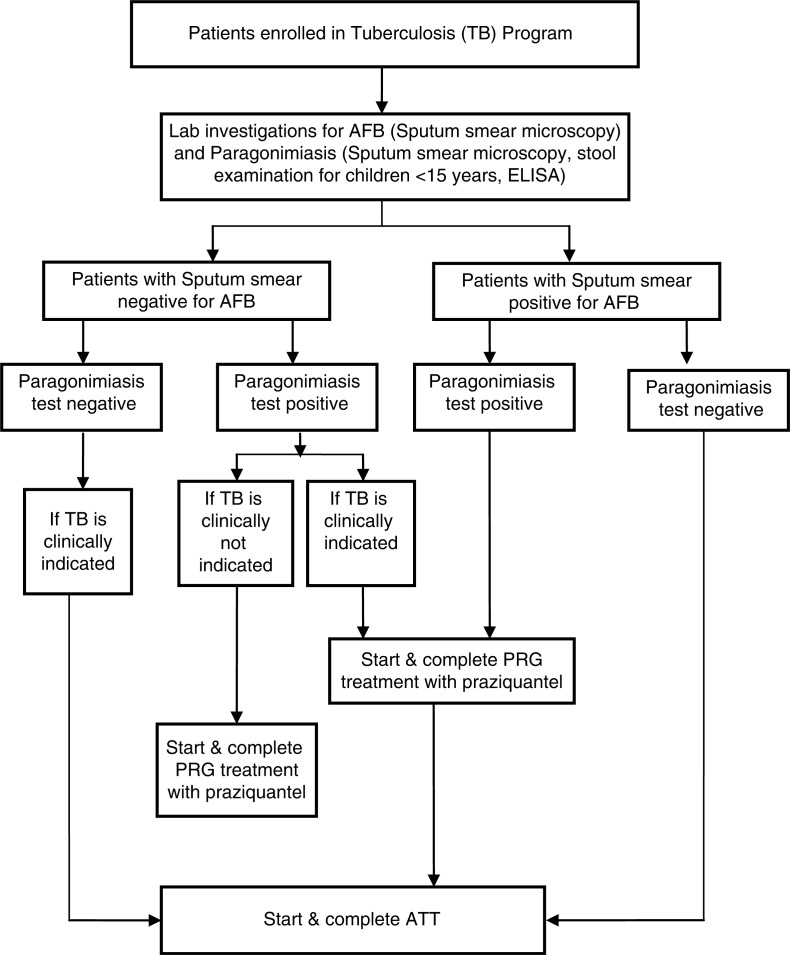
Diagnostic and treatment algorithm for tuberculosis patients co-infected with paragonimiasis. AFB: acid-fast bacilli for *Mycobacterium tuberculosis* (TB); ELISA: the enzyme-linked immunosorbent assay; PRG: paragonimiasis; ATT: anti-TB treatment.

We conducted a cross-sectional study to assess the prevalence of PRG in patients receiving TB treatment in the MSF-supported TB programme in Mon district, in collaboration with the Regional Medical Research Centre (RMRC), Dibrugarh, Assam, between November 2012 and December 2013. A diagnostic and treatment algorithm was followed (Fig. 1). Sputum smear microscopy, stool examination for children <15 years and ELISA were carried out to identify PRG and TB in patients, and appropriate treatments with anti-TB drugs and/or praziquantel were offered free of charge.

The study received approval from the MSF Ethics Review Board, Geneva, Switzerland. Informed consent was obtained from all study participants.

## Results

Ninety-six patients who had given consent were screened of whom three (3%) had pulmonary PRG, including one with HIV co-infection. Of these three PRG patients, two had smear-positive TB with no improvement in clinical condition with TB treatment; however, the third was diagnosed as smear-negative TB. Subsequent treatment with praziquantel led to substantial improvement of symptoms and eventually to cure among all three patients.

## Discussion

This study provided some limited but important evidence that PRG may be integrated into the TB diagnostic algorithms (Table 1) in PRG-endemic areas (4). We have constructed a simple diagnostic and treatment algorithm using a limited number of diagnostic tests and medicines. Praziquantel may have pharmacological interactions with TB medicines (12). In case of TB–PRG co-infection, it is preferable to treat PRG first followed by anti-TB treatment a few days later. If the diagnosis of PRG has been made after TB treatment initiation, a 4-week period may be considered before the PRG treatment initiation.

**Table 1:** Recommendations for tuberculosis (TB) programmes in paragonimiasis (PRG)-endemic areas

1.	Inquire about history of consumption of crustaceans (crab and cray fish) in presumptive TB patients
2.	Investigate for PRG and TB simultaneously including laboratory evaluation
3.	If diagnosed with TB–PRG co-infection provide treatment for PRG before anti-TB treatment
4.	Repeat laboratory evaluation for PRG, in case of no clinical improvement of TB patients on treatment or upon suspicion of TB treatment failure
5.	Consider mass triclabendazole administration in communities where cases of PRG are significantly clustered

A 2-dose/1-day regimen with triclabendazole may be preferred over the 3-day praziquantel regimen for simplicity as this may ensure higher adherence to treatment. Triclabendazole may be shipped free of charge upon application from ministries of health to the World Health Organization, while mass drug administration may be considered in communities where cases of PRG are significantly clustered (10).

## Paper context

Tuberculosis (TB) and paragonimiasis (PRG) are similar in clinical presentation; however, the modes of transmission are different. In the northeastern states of India, both TB and PRG are present but PRG is underdiagnosed. We documented the prevalence of PRG among TB patients (3/96) in the Médecins Sans Frontières (MSF)-supported TB programme in Mon district (2012–2013) using sputum smear, stool examination for children<15 years and ELISA. Our study indicates PRG may be integrated into the TB diagnostic algorithms in PRG-endemic areas.
